# LncRNA SNHG1 modulates adipogenic differentiation of BMSCs by promoting DNMT1 mediated Opg hypermethylation via interacting with PTBP1

**DOI:** 10.1111/jcmm.16982

**Published:** 2021-12-02

**Authors:** Xiao Yu, Meng‐Sheng Song, Peng‐Ze Rong, Xian‐Jun Chen, Lin Shi, Cheng‐Hao Wang, Qing‐Jiang Pang

**Affiliations:** ^1^ Department of Orthopedics HwaMei Hospital University of Chinese Academy of Sciences Ningbo China; ^2^ Ningbo Institute of Life and Health Industry University of Chinese Academy of Sciences Ningbo China; ^3^ School of Medicine Ningbo University Ningbo China

**Keywords:** DNMT1, LncRNA SNHG1, mesenchymal stem cells, osteoporosis, osteoprotegerin, PTBP1

## Abstract

Recent evidence indicates that the abnormal differentiation of bone marrow‐derived mesenchymal stem cells (BMSCs) plays a pivotal role in the pathogenesis of osteoporosis. LncRNA SNHG1 has been found to be associated with the differentiation ability of BMSCs. In this study, we aimed to elucidate the role of lncRNA SNHG1 and its associated pathway on the differentiation of BMSCs in osteoporosis. Mice that underwent bilateral ovariectomy (OVX) were used as models of osteoporosis. Induced osteogenic or adipogenic differentiation was performed in mouse BMSCs. Compared to sham animals, lncRNA SNHG1 expression was upregulated in OVX mice. Also, the in vitro expression of SNHG1 was increased in adipogenic BMSCs but decreased in osteogenic BMSCs. Moreover, overexpression of SNHG1 enhanced the adipogenic capacity of BMSCs but inhibited their osteogenic capacity as determined by oil red O, alizarin red, and alkaline phosphatase staining, while silencing of SNHG1 led to the opposite results. LncRNA SNHG1 interacting with the RNA‐binding polypyrimidine tract‐binding protein 1 (PTBP1) promoted osteoprotegerin (Opg) methylation and suppressed Opg expression via mediating DNA methyltransferase (DNMT) 1. Furthermore, Opg was showed to regulate BMSC differentiation. Knockdown of SNHG1 decreased the expressions of adipogenic related genes but increased that of osteogenic related genes. However, the knockdown of Opg partially reversed those effects. In summary, lncRNA SNHG1 upregulated the expression of DNMT1 via interacting with PTBP1, resulting in Opg hypermethylation and decreased Opg expression, which in turn enhanced BMSC adipogenic differentiation and contributed to osteoporosis.

## 1 INTRODUCTION

Osteoporosis is a systemic skeletal disorder featured by less bone mass and weakened bone strength, resulting in bone fragility and occurs when bone resorption exceeds new bone formation.^1^ Approximately 30% of females and 12% of males are affected sometime in their lives, especially among elderly and postmenopausal women.^2^ The consequences of osteoporotic fractures can be serious, including disability, reduced dependence and even death. Osteoporosis has become a major and prevalent disease worldwide and significantly increased the burden of health care.^3^ The aim of osteoporosis treatment is to restore bone homeostasis and maintain normal bone mass. Although there are a number of available agents for osteoporosis treatment in the market, concerns have been raised regarding the inherent side effects of their long‐term use, prompting the exploration of novel therapeutic targets.^4^ However, the pathophysiology of osteoporosis is multifactorial, including the imbalance between osteoblasts and osteoclasts, disrupted microarchitecture, and increased adipogenesis in the bone marrow, as well as changes in angiogenesis, oxidative stress and genetic/epigenetic factors.^5^ Recent evidence indicates that the aberrant differentiation of bone marrow‐derived mesenchymal stem cells (BMSCs) is one of the major contributing factors for the development of osteoporosis and therefore could be a potential target for the treatment of osteoporosis.^6^

Long non‐coding RNAs (lncRNAs) are a class of transcripts containing 200 or more nucleotides and have low or no capability of protein‐coding.^7^ It is conceivable that lncRNAs play important roles in gene expression on both transcriptional and posttranscriptional levels and regulating various biological functions and pathological implications, including those of the bone.^8^ Indeed, accumulating evidence suggests that various lncRNAs are involved in bone homeostasis. For example, Huang et al.^9^ demonstrated that overexpressed lncRNA H19 facilitated osteoblast differentiation of BMSCs and increased heterotopic bone formation, while knockdown of lncRNA H19 abrogated those effects. LncRNA LINC00311 has been found to induce osteoclast proliferation and inhibit osteoclast apoptosis in osteoporotic rats.^10^ A recent study also discovered that the expression of lncRNA SNHG1 could inhibit the osteogenic differentiation of BMSCs.^11^

Noncoding RNA sequences, including lncRNAs, exert their diverse functions in many cellular processes through their interactions with RNA‐binding proteins (RBPs), where RBPs play multiple roles in post‐transcriptional gene regulation.^12^ RBPs are transcription factors involved in co‐ or post‐transcriptional regulation of gene expression by affecting mRNA metabolism.^11^ The polypyrimidine tract‐binding protein 1 (PTBP1) is one of the RBPs that regulate mRNA decay stability and pre‐mRNA splicing.^13^ Apart from that, PTBP1 is also involved in the invasion of low malignancy cancer cells induced by BMSCs^14^ and is associated with the differentiation of human adipose‐derived MSCs toward definitive endoderm.^15^ To date, the potential role of PTBP1 in the differentiation of BMSCs has not been reported.

DNA methyltransferase (DNMT) 1 is an active participant of epigenetic modification and is responsible for the passive transmission of genomic methylation patterns via maintenance of DNA methylation during cell division.^16^ Osteoprotegerin (Opg) is an important regulator during BMSC differentiation and adipogenesis.^17^ As a potential target of DNMT1 and one of the key regulators of osteoclast differentiation and action, Opg functions as an inhibitor of the receptor activator of the nuclear factor kappa‐B (RANK) signalling pathway by competitively inhibiting the interaction between Opg ligand and RANK on osteoclasts and their precursors.^18^ The hypermethylation of Opg has also been found in patients with primary osteoporosis.^19^ Moreover, previous studies indicated that lncRNA SNHG1 could bind to and regulate the expression of DNMT1 in certain diseases, such as cancers.^20^, ^21^ Therefore, we hypothesized that lncRNA SNHG1 could regulate the expression of DNMT1 and subsequently influence Opg methylation status, contributing to the occurrence of osteoporosis, but it needs further determination.

The aim of the present study was to elucidate the functional role of lncRNA SNHG1 in osteoporosis pathogenesis as well as the underlying mechanistic pathways.

## 2 MATERIALS AND METHODS

### 2.1 Animals

All animals were housed and handled in accordance with the Guide for the care and use of laboratory animals. All animal studies were approved by the Experimental Animal Ethics Committee of Ningbo University (2020‐106). The mouse model of oestrogen deficiency‐induced osteoporosis was established as previously described.^22^ Female C57BL/6 mice weighing 20–25 g at 11–12 weeks of age were housed under standard conditions with a 12 h light/dark cycle. The temperature and the humidity were controlled at 20–25°C and 40%–70% respectively. Mice had free access to standard chow and water. Before randomization, all animals were acclimatized to the environment for at least 5 days. Subsequently, mice were randomized to undergo bilateral ovariectomy (OVX) or sham surgery (sham). After 8 weeks, the mouse femurs were collected after euthanasia and fixed with paraformaldehyde for 48 h. Blood samples were collected and bone tissues were harvested. Samples were then frozen immediately in liquid nitrogen and stored at −80°C for further experiments.

### 2.2 Clinical tissues collection

In this study, six pairs of bone tissues were harvested from osteoporotic patients and non‐osteoporotic patients (controls) underwent routine therapeutic surgery in our hospital and instantly stored at −80°C for subsequent analyses. The experimental protocols were reviewed and approved by the Ethics Committee of HwaMei Hospital, University of Chinese Academy of Sciences (PJ‐NBEY‐KY‐2019‐081‐01) and all of the methods in this study were in accordance with the approved guidelines. All participating patients gave written informed consent before any study procedures occurred.

### 2.3 Cell culture and treatment

Mouse BMSCs (MUBMX‐01001) were obtained from Cyagen Inc., and cultured at 37°C, 5% CO_2_ in Alpha‐MEM medium contained 10% fetal bovine serum (Gibco), 2 mmol/l L‐glutamine, 100 U/ml penicillin, and 100 μg/ml streptomycin. BMSCs were then treated with either osteogenic differentiation‐inducing medium (formulated by adding 10 mM β‐sodium glycerophosphate, 0.1 μM dexamethasone, and 50 mg/ml ascorbic acid to the growth media) or adipogenic differentiation‐inducing medium (formulated by adding 1% double antibody, 10 mM 3‐isobutyl‐1‐methylxanthine, 10 mM indomethacin, 10 nM dexamethasone to high sugar MEM media) to induce osteogenic or adipogenic differentiation respectively.^23^ Alizarin red S (ARS) staining and oil red O (ORO) staining were performed to detect osteogenic and adipogenic differentiation after culture with a corresponding inducing medium. BMSCs treated with growth media were served as controls. After induction, cells were harvested for further analysis. For 5‐Aza‐2′‐deoxycytidine (5‐Aza) treatment, cells were treated with 10 µM 5‐Aza (Sigma) for 96 h and the medium was replaced every 24 h.

### 2.4 Plasmid construction and transfection

The full‐length lncRNA SNHG1 sequences were amplified by PCR and then inserted into the pcDNA 3.1 vector (Life Technologies) to establish a vector overexpressing SNHG1 (pcDNA‐SNHG1) according to the manufacturer's instructions. The empty vector was served as a negative control (NC). Using the same method as above, PTBP1 and Opg were cloned and termed as pcDNA‐PTBP1 and pcDNA‐Opg respectively. Short‐hairpin (sh) RNAs directed against lncRNA SNHG1, PTBP1, Opg and sh‐negative control (sh‐NC) were obtained from GenePharma. Cell transfection was performed using Lipofectamine 3000 (Life Technologies). Briefly, BMSCs grown to 70% confluence in alpha‐MEM medium were incubated with Lipofectamine 3000 reagent and a pcDNA or an sh‐RNA for 48 h before culturing in adipogenic or osteogenic induction medium. For in vivo transfection, lenti‐sh‐SNHG1 and/or sh‐Opg were injected into the circulation via the tail vein. Mice were finally euthanized after continuous injection for 3 weeks.

### 2.5 Haematoxylin and eosin staining

The mouse femurs were fixed in phosphate‐buffered 10% paraformaldehyde for 24 h followed by decalcification with 8% formic acid at 4°C under continuous shaking. Dehydrated tissues were then embedded in paraffin and sliced into 5 µm sections for haematoxylin and eosin (HE) staining. Haematoxylin and eosin staining was conducted according to routine protocols. Briefly, after deparaffinization and rehydration, 5 μm longitudinal sections were stained with haematoxylin solution for 5 min followed by 5 dips in 1% acid ethanol (1% HCl in 70% ethanol) and then rinsed in distilled water. Then the sections were stained with eosin solution for 3 min and followed by dehydration with graded alcohol and clearing in xylene. At least 5 fields were selected on a random basis and photographed under light microscopy (Nikon) for histological evaluation.

### 2.6 ORO and ARS staining

Oil red O staining and ARS staining were performed 14 days after osteogenic and adipogenic differentiation of BMSCs as previously described.^23^ The cell culture medium was discarded and the cells were washed three times with phosphate‐buffered saline (PBS; Solarbio). The cells were fixed in 4% paraformaldehyde at room temperature (RT) for 20 min and then stained with ORO (Cyagen Biosciences) or ARS (Cyagen Biosciences) for 30 min, followed by washing with deionized water for three times according to the manufacturer's instructions. After washing, lipid droplets of cells were photographed with a phase‐contrast microscope. For quantification of mineralization, ARS staining was solubilized by 100 mmol/l cetylpyridinium chloride (Sigma). After incubation for 1 h, the absorbance was measured spectrophotometrically at 570 nm.

### 2.7 Immunofluorescent staining

Immunofluorescence (IF) staining was performed as follows. Cells were washed by PBS and fixed by using 4% paraformaldehyde at RT for 20 min. Then, cells were rinsed with PBS three times and permeabilized in 0.3% Tween‐100 (Biosharp) at RT for 1 h. After blocking with an appropriate volume of available goat serum (Boster Biological Technology) for 30 min and washing three times, the samples were incubated with primary anti‐Ppar‐γ antibody (ab59256, dilution 1:200; Abcam) overnight at 4°C. The sample was washed with PBS three times followed by incubation at RT for 1 h with the fluorescein‐labelled corresponding secondary antibody. Nuclei were counterstained with 4, 6‐diamidino‐2‐phenylindole dihydrochloride (DAPI) (D8417; Sigma). Photos were captured with an IX73 microscope (Olympus) to demonstrate the adipogenic differentiation.

### 2.8 Alkaline phosphatase (ALP) staining and activity

Alkaline phosphatase staining was performed as described previously on day 14 following osteogenic differentiation of BMSCs.^24^ In brief, cells were washed with PBS and fixed by using 4% paraformaldehyde for 10 min and then incubated in ALP solution (CWBIO) for staining for 20 min protected from light at RT. After three times wash, calcium mineralization pictures were obtained using an inverted optical microscope. After incubation with 10 mM p‐nitrophenyl phosphate (Sigma) for 15 min, ALP activity was calculated at 420 nm by a microplate reader.

### 2.9 Fluorescence in situ hybridization (FISH) analysis

Biotin‐labelled sense and antisense SNHG1 probes and the corresponding control oligo for FISH analysis were obtained from GenePharma and FISH assays were performed as previously described.^25^ For immobilization, cells were treated with 4% paraformaldehyde for 20 min at RT. After washing with PBS, the cells were prehybridized with a hybridization solution. Then, the cells were incubated with the SNHG1 probes overnight at 37°C. An anti‐Biotin‐Cy3 antibody (C5585; Sigma) was used for signal detection. Cell nuclei were counterstained with DAPI to assess the nuclear morphology. Samples were incubated with diluted DAPI solution at a concentration between 0.5 µg/ml in PBS for 5 min at RT in the dark. The immunofluorescence was measured by using an IX73 microscope (Olympus).

### 2.10 RNA pull down assay

The biotinylated RNA pull‐down assay was performed as previously described in order to determine the interaction between lncRNA SNHG1 and PTBP1.^26^ The biotin‐labelled RNAs used in the assay were synthesized by in vitro transcription with the Biotin RNA Labeling Mix (Roche) and T7 RNA polymerase. In brief, cells were lysed in immunoprecipitation (IP) buffer (20 mM Tris‐HCl, pH 8.0, 200 mM NaCl, 1 mM EDTA, 1 mM EGTA, 0.5% Triton X‐100, 0.4 U/μl RNasin) to a final cell concentration of 5 × 10^6^ cells/ml. The assay was conducted by incubating the whole‐cell lysates with biotinylated RNAs for 30 min at RT. RNA‐protein complexes were then isolated by streptavidin magnetic beads (Thermo Scientific). RNA‐protein complexes were incubated with streptavidin magnetic beads for 30 min at RT on a roller. The magnetic beads were separated in a magnetic stand, and the samples were eluted after incubation with elution buffer at 37°C on a shaker for 30 min. The precipitated components were separated using dodecyl sulfate‐polyacrylamide gel electrophoresis (SDS‐PAGE) and visualized by western blot.

### 2.11 The RNA immunoprecipitation assay

The RNA immunoprecipitation (RIP) assay was conducted by using a commercial EZ‐Magna RIP™ Kit (Millipore) according to the manufacturer's instructions. In brief, cells (5 × 10^6^) were treated with RIP lysis buffer at 4°C for 30 min and then whole‐cell lysates were incubated with RIP buffer containing magnetic beads conjugated to antibodies against Ago2 (Millipore) or anti‐PTBP1 antibody (ab133734; Abcam) or anti‐SNRP70 antibody (ab51266; Abcam) or immunoglobulin (Ig) G (Abcam) for 2 h at RT with gentle shaking. The coprecipitated RNAs associated with PTBP1 were extracted with the Trizol reagent (Life Technologies) and analysed by quantitative real‐time PCR (qPCR). Enrichment associated with SNRP70 and species‐matched normal IgG served as positive and negative RIP controls respectively. Total RNA was considered as input controls.

### 2.12 qPCR assay

Animal bone tissues were harvested from OVX and sham mice. Human bone samples were collected from patients diagnosed with osteoporosis and control patients. Total RNA was extracted from BMSCs and bone tissues by using Trizol (Life Technologies) method. Cells or tissues were homogenized in Trizol and extracted with chloroform (0.2 ml/ml Trizol). Isopropanol (0.5 ml/ml Trizol) was used to precipitate the RNA from its aqueous phase. RNA was pelleted by centrifugation at 7500 *g* and 4°C for 5 min, washed in 75% ethanol, centrifuged again, air‐dried for 10 min and dissolved in 200 μl HPLC water. A NanoDrop spectrophotometer (Thermo Scientific) was used for RNA concentration measurement and an Agilent Bioanalyzer RNA 6000 Nano kit (Agilent Technologies) was used for RNA quality determination. Extracted RNA was then reverse transcribed to first‐strand cDNA by using the iScript™ cDNA synthesis kit (Bio‐Rad). qPCR analysis was carried out by using an UltraSYBR Mixture kit (Thermo Scientific) on an Applied Biosystems 7500 Fast Dx Real‐Time PCR System (Life Technologies). The primers used in the qPCR assay were as follows: human SNHG1 (Forward: CCACCTTCTGTTCCCGTCAT, Reverse: GACAGCCAGTCCTCAAGGGA); mouse SNHG1 (Forward: TCCTTGTTCGGGGTTTGAGG, Reverse: ACAGCACCCTGACTACAAGC); Opg (Forward: GAGTGTGAGGAAGGGCGTTA, Reverse: GTGCTGCAGTTCGTGTGTTT); Runt‐related transcription factor 2 (Runx2) (Forward: AGATGGGACTGTGGTTACCG, Reverse: GGACCGTCCACTGTCACTTT); ALP (Forward: CATTCCCATGTCTTCACCTTTG, Reverse: TCCTCTTGTTCCGTTCACATG); Bone morphogenetic protein 4 (Bmp4) (Forward: CATTCCCATGTCTTCACCTTTG, Reverse: TCCTCTTGTTCCGTTCACATG); Bone gamma‐carboxyglutamic acid‐containing protein (Bglap) (Forward: CATTCCCATGTCTTCACCTTTG, Reverse: TCCTCTTGTTCCGTTCACATG); Peroxisome proliferator‐activated receptor gamma (Ppar‐γ) (Forward: AAGAGCTGACCCAATGGTTG, Reverse: ACCCTTGCATCCTTCACAAG); Fatty acid binding protein 4 (Fabp4) (Forward: CGTAAATGGGGATTTGGTCA, Reverse: TCGACTTTCCATCCCACTTC); CCAAT enhancer binding protein alpha (Cebpa) (Forward: GTGGACAAGAACAGCAACGA, Reverse: CCTTGACCAAGGAGCTCTCA); PTBP1 (Forward: CAGAGGACGACCTCAAGAGC, Reverse: GGTGGACTTGGAAAAGGACA); DMNT1 (Forward: GATCCATTTGGCTGGTGTCT, Reverse: CATGGCATTCTCCTTGTCCT); Collagen type 1 alpha 1 (Col1a1) (Forward: TGACTGGAAGAGCGGAGAGT, Reverse: GTTCGGGCTGATGTACCAGT); human GAPDH (Forward: CCAGGTGGTCTCCTCTGA, Reverse: GCTGTAGCCAAATCGTTGT); mouse GAPDH (Forward: AGCCCAAGATGCCCTTCAGT, Reverse: CCGTGTTCCTACCCCCAATG). GAPDH served as internal references. During the experimental setup for in‐house validation, the expressions of above target genes did not alter in cells transfected with empty pcDNA 3.1 vector or sh‐NC, when compared with untransfected cells. Therefore, the empty pcDNA 3.1 vector and the sh‐NC would serve as a control for subsequent transfection studies when gene expression was analysed. The relative expression value of target genes was calculated by applying the relative quantification 2^−∆∆Ct^ method to measure fold‐change. All measurements were repeated three times.

### 2.13 Quantitative methylation‐specific PCR

Quantitative methylation‐specific PCR (MSP) was designed to specifically amplify bisulphite converted DNA target.^27^ By targeting CpG‐rich regions within the Opg regions, the primers of Opg can specifically recognize the bisulphite‐treated methylated and unmethylated DNA. Therefore, we performed MSP to measure the methylation status of Opg. The methylation‐specific Opg primers were: Forward: TTCGGATTATGGTTGCATC; Reverse: GCAAACTCTATAATTTCGCG. Genomic DNA were amplified at conditions: 95°C for 5 min, followed by 40 cycles of 95°C for 30 s, 57°C for 30 s and 72°C for 30 s, with a final extension of 72°C for 5 min. Products of MSP were finally visualized on 2% ethidium bromide stained agarose gels. The degree of DNA methylation was estimated as the unmethylated primer cycle threshold minus the methylated primer cycle threshold for each amplicon.

### 2.14 Subcellular fractionation

The separation of the nuclear and cytosolic fractions was performed using the NE‐PER Nuclear and Cytoplasmic Extraction Reagents (Thermo Scientific) according to the manufacturer's instructions. Briefly, cells were harvested and incubated with ice‐cold CER I on ice for 10 min. Then, ice‐cold CER II was added, followed by incubation on ice for another 5 min. After centrifugation at about 4000 *g* for 5 min, the supernatant was transferred to a new tube. This was the cytoplasmic fraction. The pellet was suspended with ice‐cold NER and vortexed on the highest setting for 15 s every 10 min for a total of 40 min. After centrifugation at about 4000 *g* for 5 min, the supernatant was collected as the nuclear fraction. Total RNA was isolated from cytoplasmic and nuclear extracts. qPCR was performed to assess the relative proportion in the nuclear and cytoplasmic fractions. U6 and GAPDH were served as nuclear and cytoplasmic markers, respectively.

### 2.15 Western blotting

Whole‐cell and tissue lysates were prepared by using the radio‐immunoprecipitation assay (RIPA) buffer supplemented with protease inhibitors (Roche) at 4°C. A Direct Detect Spectrometer (Merck) was used to measure the concentration of protein. Twenty micrograms of protein was separated by 10% sodium SDS‐PAGE in reducing conditions and then transferred onto polyvinylidene difluoride (PVDF) membranes through electroblotting. After blocked with 5% non‐fat milk, the membranes were incubated with the diluted primary rabbit antibodies from Abcam against PTBP1 antibody (ab133734; dilution 1:20000) Opg (ab183910, dilution 1:1000), Ppar‐γ (ab59256, dilution 1:1000), Fabp4 (ab92501, dilution 1:2000), Runx2 (ab23981, dilution 1:1000), and β‐actin (ab179467, dilution 1:5000) overnight at 4°C. Membranes were then incubated with species‐matched secondary antibodies for 2 h at RT. The immunoreactive bands were detected using enhanced chemiluminescence substrate and captured by a BioRad Chemidoc MP system (Bio‐Rad). The fold changes of the target proteins were normalized to the level of housekeeping protein β‐actin.

### 2.16 Statistical analysis

In all in vitro studies, experiments were conducted at least three replications with at least three independent samples each time. In in vivo studies there were six mice per group for all animal studies. The data were presented as mean ± standard deviation (SD). Significance was determined using Student's *t*‐test or one‐way ANOVA with post‐hoc analysis. Statistical analysis was carried out by using IBM SPSS Statistics for Windows, Version 23.0 (IBM). A *p*‐value of <0.05 was considered statistically significant.

## 3 RESULTS

### 3.1 LncRNA SNHG1 is associated with BMSC differentiation in osteoporosis

**FIGURE 1:**
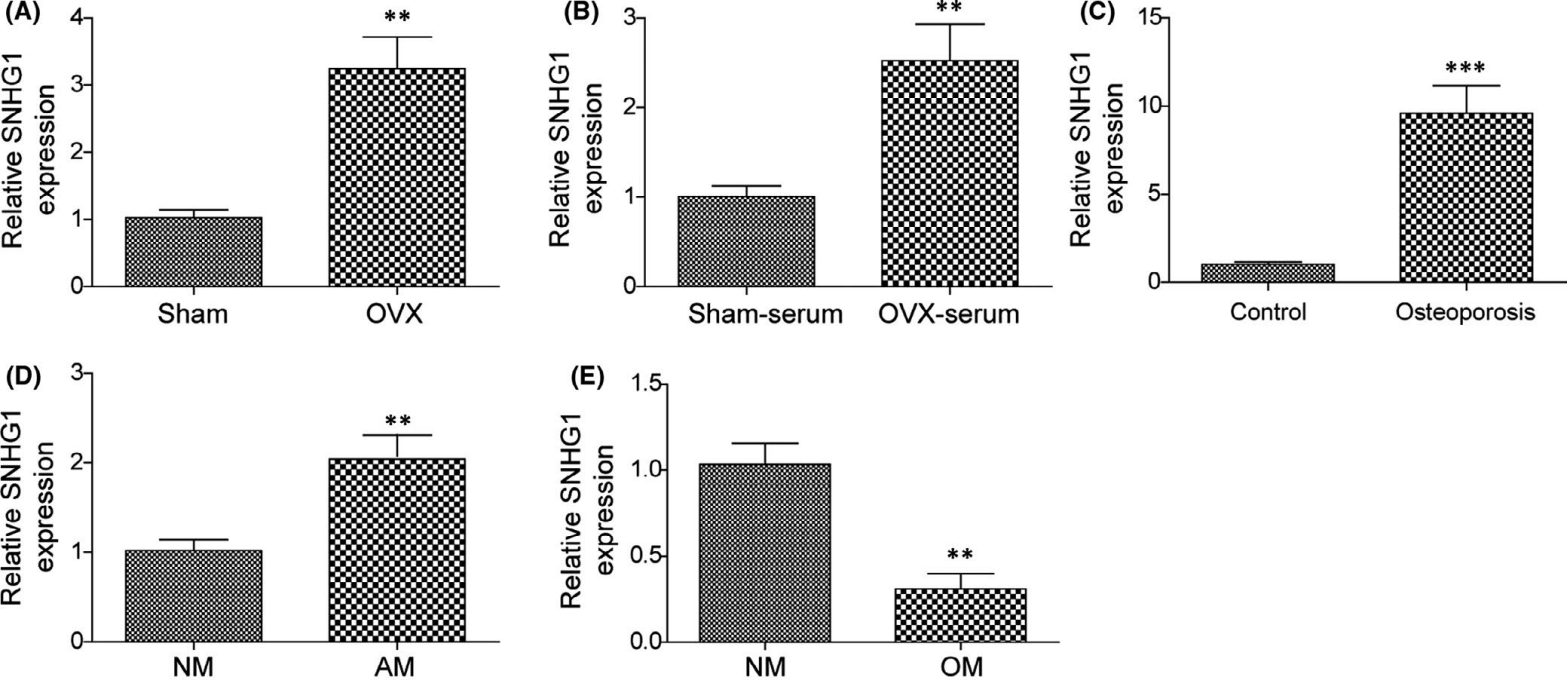
LncRNA SNHG1 is associated with BMSC differentiation in osteoporosis. LncRNA SNHG1 expressions of the following samples were determined by qPCR: (A, B) Bone tissues and blood serums of OVX and shame mice; (C) Bone tissues of osteoporosis and control patients; (D) Adipogenic inducer and growing media treated BMSCs; (E) Osteogenic inducer and growing media treated BMSCs. AM, adipogenic induced medium; NM, normal growth medium; OM, osteogenic induced medium. Comparisons were conducted using Student's *t*‐test. **p < 0.01; ***p < 0.001

In order to elucidate the role of lncRNA SNHG1 and its relationship with BMSC differentiation in osteoporosis, we first looked into the expression of lncRNA SNHG1 in in vivo mouse models and osteoporosis patients. The qPCR revealed that the expression of lncRNA SNHG1 was significantly upregulated in bone tissues and blood serums of OVX mice, when compared to sham control mice (Figure 1A,B). Similarly, we observed increased lncRNA SNHG1 expressions in tissues of osteoporosis patients than those of control patients (Figure 1C). We further investigated lncRNA SNHG1 expressions in osteogenic inducer or adipogenic inducer stimulated BMSCs. On the 14th day of induction, the induction of adipogenic differentiation markedly enhanced the expression of lncRNA SNHG1 in BMSCs while the expression of lncRNA SNHG1 was notably downregulated in cells treated with osteogenic inducing media (Figure 1D,E). These results indicate that the expression of lncRNA SNHG1 is associated with the BMSC differentiation in osteoporosis.

### 3.2 LncRNA SNHG1 enhances BMSC adipogenic differentiation but inhibits BMSC osteogenic differentiation

**FIGURE 2:**
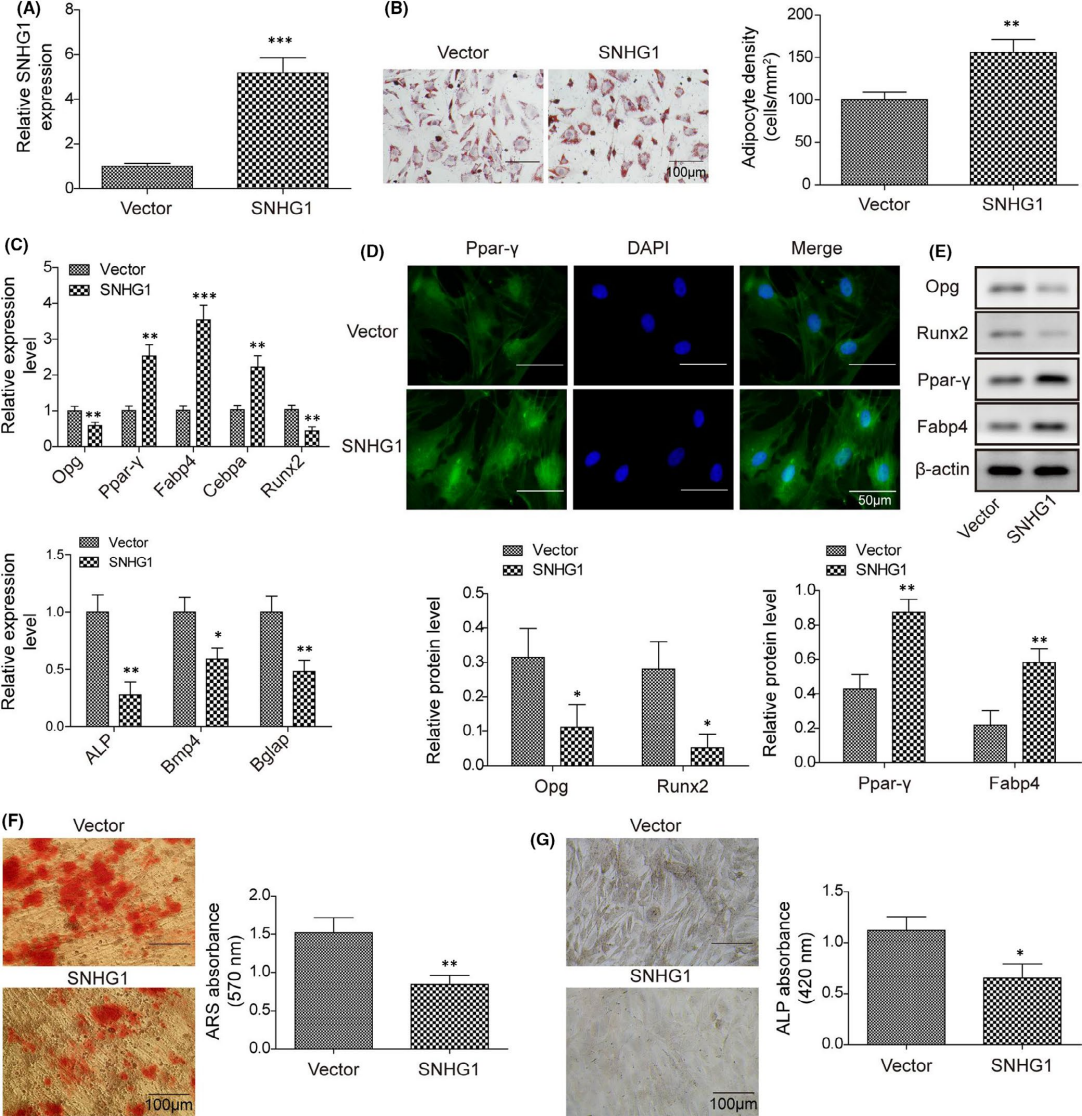
LncRNA SNHG1 enhances BMSC adipogenic differentiation but inhibits BMSC osteogenic differentiation. BMSCs were transfected with pcDNA‐SNHG1 or empty vector. (A) LncRNA SNHG1 expression of BMSCs transfected with pcDNA‐SNHG1 or empty vector; (B) Calculated adipocyte density after ORO staining with represented images; (C) The mRNA expressions of Opg, Runx2, Ppar‐γ, Fabp4, ALP, Bmp4, Bglap and Cebpa by qPCR; (D) Immunofluorescence staining for Ppar‐γ; (E) The protein levels of Opg, Runx2, Ppar‐γ and Fabp4 by western blot; (F) ARS absorbance after ARS staining with represented images; (G) ALP absorbance after ALP staining with represented images. Comparisons were conducted using Student's *t*‐test. *p < 0.05; **p < 0.01; ***p < 0.001

We explored the role of lncRNA SNHG1 in BMSC differentiation in transfected BMSCs. The expression of lncRNA SNHG1 was significantly upregulated in cells transfected with pcDNA‐SNHG1, when compared with those transfected with empty vector, confirming the successful transfection of pcDNA‐SNHG1 (Figure 2A). Results of the ORO staining indicated that the adipogenic differentiation ability of BMSCs transfected with pcDNA‐SNHG1 was enhanced when treated with adipogenic inducing medium, resulting in increased lipid droplet formation and adipocyte density (Figure 2B). Whereas the ARS staining showed that the osteogenic differentiation ability of pcDNA‐SNHG1 transfected BMSCs was lower than that transfected with empty vector when treated with osteogenic inducing medium, and fewer mineralized nodules were formed (Figure 2F). We further examined the expressions of osteogenesis‐related genes (Opg, Runx2, ALP, Bmp4, Bglap) and adipogenesis‐related genes (Ppar‐γ, Fabp4, Cebpa) in the corresponding group to confirm our observations. Consistent with the staining results, the gene and protein expressions of adipogenesis‐related genes in BMSCs with SNHG1 overexpressed were increased, while the expressions of osteogenesis‐related genes were decreased (Figure 2C,E). Moreover, overexpression of lncRNA SNHG1 increased Ppar‐γ fluorescence signals in BMSCs with immunofluorescence staining (IF) (Figure 2D), but inhibited ALP activity with a smaller and less intense stained area after ALP staining (Figure 2G). Taken together, our observations indicate that lncRNA SNHG1 regulates BMSC differentiation by enhancing adipogenic differentiation but inhibiting osteogenic differentiation.

### 3.3 Silencing lncRNA SNHG1 suppresses BMSC adipogenic differentiation but promotes BMSC osteogenic differentiation

**FIGURE 3:**
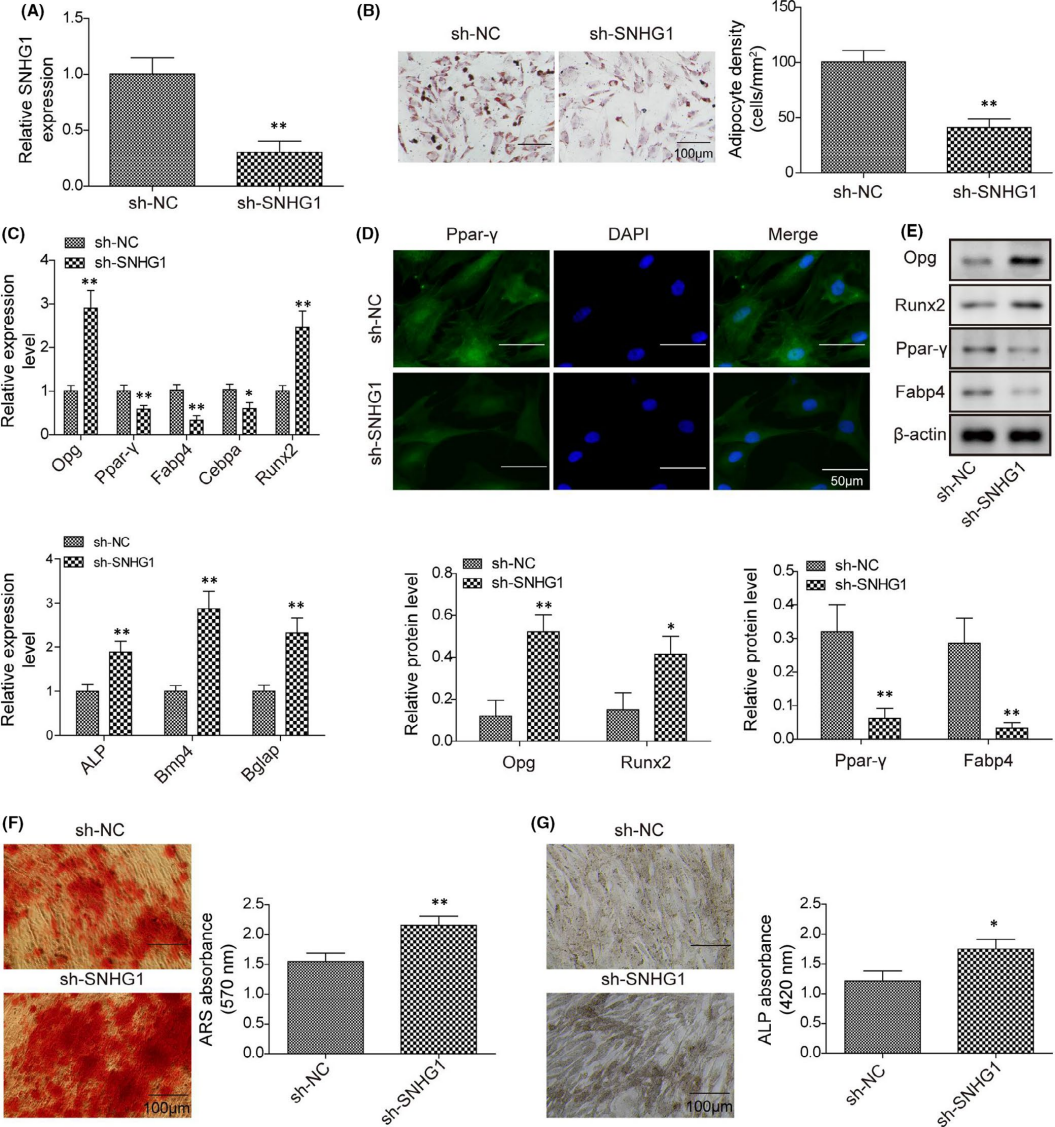
Silencing lncRNA SNHG1 suppresses BMSC adipogenic differentiation but promotes BMSC osteogenic differentiation. BMSCs were transfected with sh‐SNHG1 or sh‐NC. (A) LncRNA SNHG1 expression of BMSCs transfected with sh‐SNHG1 or sh‐NC; (B) Calculated adipocyte density after ORO staining with represented images; (C) The mRNA expressions of Opg, Runx2, Ppar‐γ, Fabp4, ALP, Bmp4, Bglap and Cebpa by qPCR; (D) Immunofluorescence staining for Ppar‐γ; (E) The protein levels of Opg, Runx2, Ppar‐γ and Fabp4 by western blot; (F) ARS absorbance after ARS staining with represented images; (G) ALP absorbance after ALP staining with represented images. Comparisons were conducted using Student's *t*‐test. *p < 0.05; **p < 0.01

We further explored the impact of lncRNA SNHG1 silencing on BMSC differentiation in BMSCs transfected with sh‐SNHG1. Transfection with sh‐SNHG1 successfully repressed the expression of lncRNA SNHG1 in BMSCs in repeated experiments, when compared with transfection with sh‐NC (Figure 3A). The osteogenic or adipogenic differentiation induction in BMSCs, as well as a series of staining, were sequentially performed. The gene and protein expressions of osteogenesis‐ and adipogenesis‐related genes were also examined. As expected, lncRNA SNHG1 silencing significantly suppressed the adipogenic differentiation ability of BMSCs presented as reduced lipid droplet formation but enhanced the osteogenic differentiation ability presented as increased mineralized nodule formation and ALP activity when treated with adipogenic and osteogenic inducing medium, respectively (Figure 3B,F,G). Meanwhile, the gene and protein expressions of adipogenesis‐related genes were decreased, while the expressions of osteogenesis‐related genes were increased in sh‐SNHG1 transfected BMSCs (Figure 3C,D,E). These findings support the role of lncRNA SNHG1 in regulating BMSC differentiation.

### 3.4 LncRNA SNHG1 interacts with PTBP1 and promotes the expression of PTBP1

**FIGURE 4:**
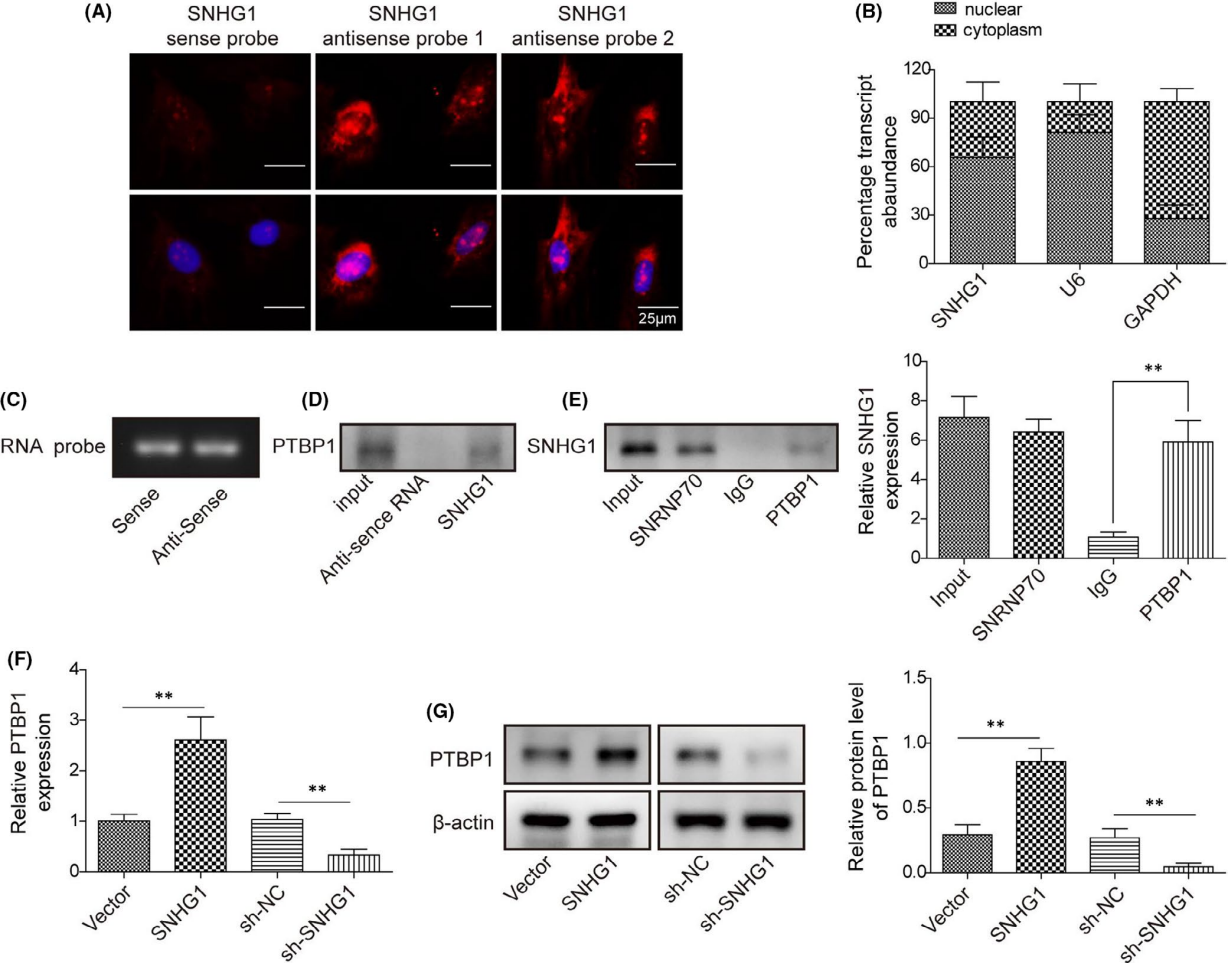
LncRNA SNHG1 interacts with PTBP1 and promotes the expression of PTBP1. (A) FISH of SNHG1 (red) in BMSCs. DAPI stained images are blue; (B) Fractionation of SNHG1 in MSCs followed by qPCR. U6 and GAPDH served as nuclear and cytoplasmic control respectively; (C) Synthesized biotinylated sense and antisense SNHG1 probes; (D) RNA pull‐down followed by western blotting. BMSC lysates were incubated with sense or antisense biotin‐labelled SNHG1 probes. After pull‐down, the recruited PTBP1 to probes was examined by western blot; (E) RIP assay to detect the interactions between PTBP1 and SNHG1 using an anti‐PTBP1 antibody. SNRP70 or normal IgG served as positive and negative controls, respectively. (F) The mRNA expression of PTBP1 by qPCR in BMSCs transfected with pcDNA‐SNHG1/empty vector or sh‐SNHG1/sh‐NC; (G) The protein level of PTBP1 by western blot in BMSCs transfected with pcDNA‐PTBP1/empty vector or sh‐ PTBP1/sh‐NC. Comparison between groups was done using Student's *t*‐test or one‐way ANOVA with post‐hoc analysis where appropriate. **p < 0.01

In order to better understand the function and mechanism of lncRNA SNHG1, the FISH and qPCR were conducted to examine specific subcellular localization and cell fractionation of lncRNA SNHG1 in cells cultured in normal medium. The FISH showed that lncRNA SNHG1 was widely localized in the cell nuclear (Figure 4A). Consistently, the qPCR revealed that lncRNA SNHG1 largely displayed a nuclear distribution (>60%) (Figure 4B). To determine the potential interaction of lncRNA SNHG1 with PTBP1, we performed RNA pull‐down assays in BMSCs using in vitro synthesized biotinylated sense and antisense SNHG1 RNAs (Figure 4C). After incubation with BMSC extracts, western blot showed that PTBP1 was identified as a binding partner of lncRNA SNHG1 (Figure 4D). Alternatively, the RIP assay was performed using a PTBP1 antibody to detect lncRNA SNHG1 in the PTBP1 precipitates. We found that PTBP1 antibody significantly precipitated lncRNA SNHG1 compared with the anti‐IgG control (Figure 4E), further confirming the interaction between lncRNA SNHG1 and PTBP1. To determine the biological effect of lncRNA SNHG1 and PTBP1 interaction, we examined the expression of PTBP1 in lncRNA SNHG1 overexpressed or silenced BMSCs. Overexpression of lncRNA SNHG1 increased the gene and protein expressions of PTBP1 while silencing of lncRNA SNHG1 reduced the expression of PTBP1 (Figure 4F,G). These results suggest that lncRNA SNHG1 interacts with PTBP1 and promotes the expression of PTBP1.

### 3.5 LncRNA SNHG1 interacts with PTBP1 and promotes the expression of DNMT1

**FIGURE 5:**
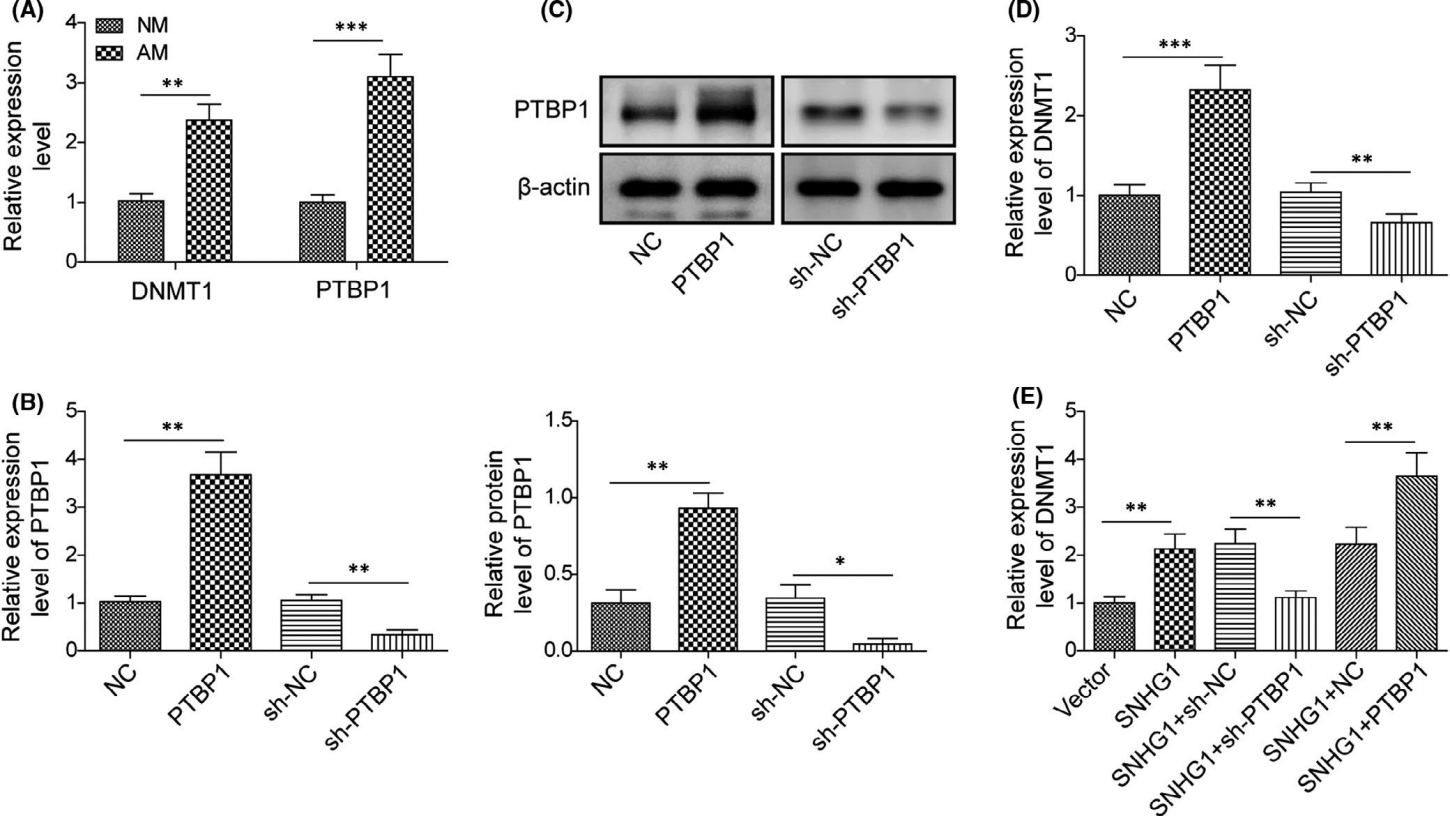
LncRNA SNHG1 interacts with PTBP1 and promotes the expression of DNMT1. (A) The mRNA expressions of PTBP1 and DNMT1 by qPCR in BMSCs treated with adipogenic differentiation inducer for 14 days; (B, C) The mRNA expression of PTBP1 by qPCR; (C) The protein level of PTBP1 by western blot; (D) The mRNA expression of DNMT1 by qPCR; (E) The mRNA expression of DNMT1 by qPCR in various groups with different transfection regimens. NM, normal growth medium; AM, adipogenic induced medium. Comparisons were conducted using Student's *t*‐test or one‐way ANOVA with post‐hoc analysis where appropriate. *p < 0.05; **p < 0.01; ***p < 0.001

Previous studies suggested that RNA‐binding proteins could affect DNMT stability and regulate DNA methylation.^28^, ^29^ We were particularly interested in DNMT1, which is one of the key enzymes maintaining DNA methylation.^30^ In order to illustrate the potential roles of PTBP1 and DNMT1 in BMSC differentiation, the gene expressions of PTBP1 and DNMT1 were examined in BMSCs treated with adipogenic differentiation inducing media for 14 days. Interestingly, treating with adipogenic inducer for 14 days significantly upregulated the expressions of both PTBP1 and DNMT1 in BMSCs (Figure 5A). We then tested the regulatory effect of PTBP1 on DNMT1 in BMSCs transfected with pcDNA‐PTBP1, sh‐PTBP1 or their corresponding negative controls. Both qPCR and western blot showed that PTBP1 positively regulated the expression of DNMT1 (Figure 5B–D). Notably, transfection with pcDNA‐SNHG1 upregulated DNMT1 expression, which was more remarkable when co‐transfection with pcDNA‐SNHG1 and pcDNA‐PTBP1, while silencing of PTBP1 partially reversed the effect of lncRNA SNHG1 on DNMT1 (Figure 5E), indicating that lncRNA SNHG1 promotes the expression of DNMT1 via interacting with PTBP1.

### 3.6 LncRNA SNHG1 enhances Opg methylation to suppress the expression of Opg

**FIGURE 6:**
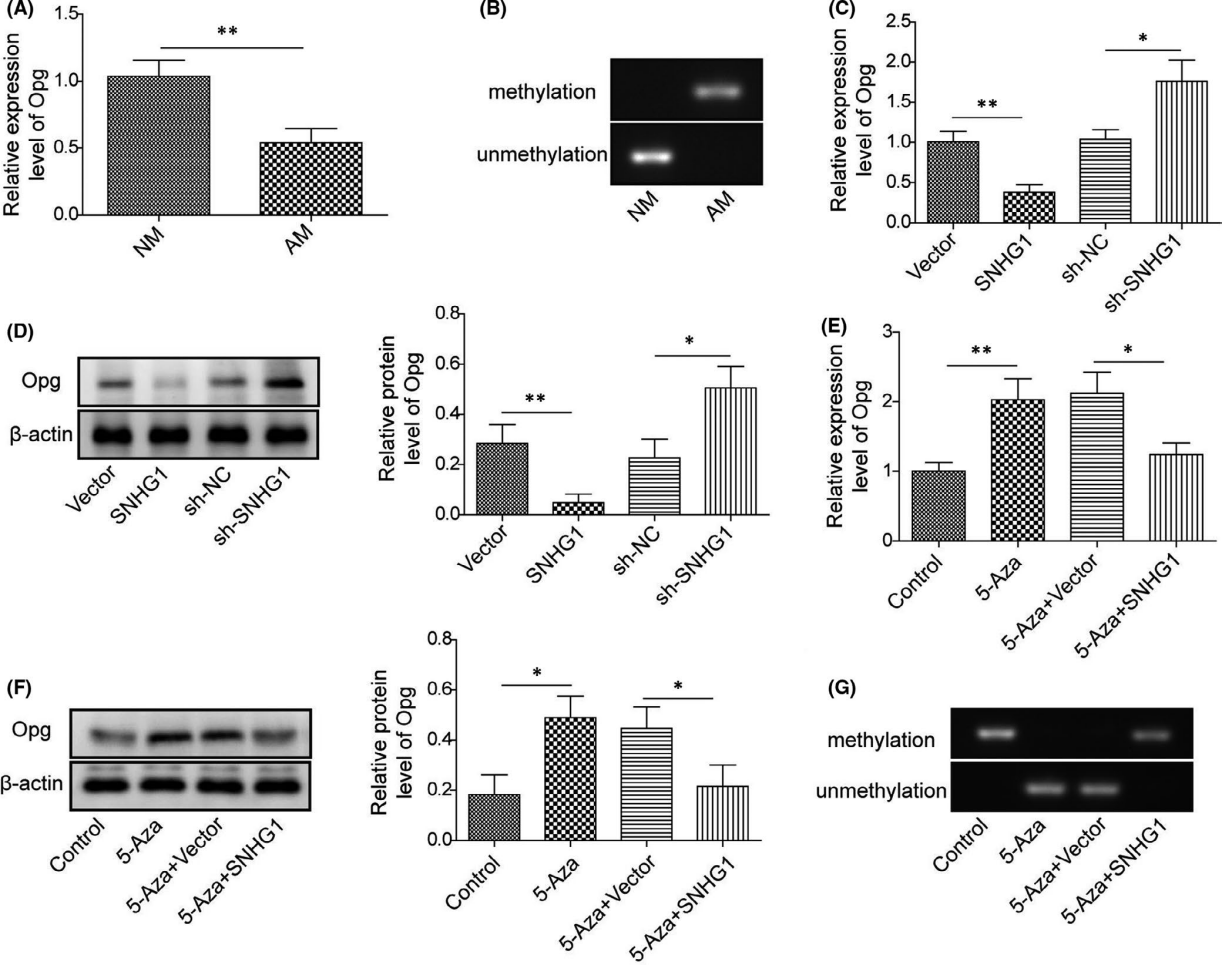
LncRNA SNHG1 enhances Opg methylation to suppress the expression of Opg. (A) The mRNA expression of Opg by qPCR in BMSCs treated with adipogenic differentiation inducer for 14 days; (B) MSP analysis to detect the methylation status of Opg; (C) The mRNA expression of Opg by qPCR in BMSCs transfected with pcDNA‐SNHG1/empty vector or sh‐SNHG1/sh‐NC; (D) The protein level of Opg by western blot in BMSCs transfected with pcDNA‐SNHG1/empty vector or sh‐SNHG1/sh‐NC; (E, F) The mRNA expression and protein level of Opg in pcDNA‐SNHG1 or empty vector transfected BMSCs after 5‐Aza treatment for 96 h; (G) MSP analysis to detect the methylation status of Opg in pcDNA‐SNHG1 or empty vector transfected MSCs after 5‐Aza treatment for 96 h. NM, normal growth medium; AM, adipogenic induced medium. Comparisons were conducted using Student's *t*‐test or one‐way ANOVA with post‐hoc analysis where appropriate. *p < 0.05; **p < 0.01

We further examined whether the effects of lncRNA SNHG1 in BMSC adipogenic differentiation were associated with the regulation of Opg methylation in BMSCs treated with adipogenic inducing media. qPCR and MSP showed that treatment with adipogenic inducer for 14 days suppressed Opg expression but promoted Opg methylation in BMSCs (Figure 6A,B). As for the effect of lncRNA SNHG1, SNHG1 overexpression exhibited significant inhibitory effects on the gene and protein expressions of Opg, while SNHG1 silencing enhanced the expression of Opg (Figure 6C,D). Moreover, treatment with the DNA demethylating agent 5‐Aza increased the expression levels of Opg, while SNHG1 overexpression abrogated those effects (Figure 6E,F). DNA methylation analysis by MSP confirmed that 5‐Aza substantially suppressed Opg methylation, whereas overexpression of SNHG1 promoted DNA methylation of Opg (Figure 6G). The inverse relationship we observed between the methylation status and the gene expression suggests that Opg methylation is evidently repressing Opg expression. Nevertheless, lncRNA SNHG1 potentially enhances Opg methylation and therefore downregulates the expression of Opg.

### 3.7 Opg regulates cell differentiation of BMSCs

**FIGURE 7:**
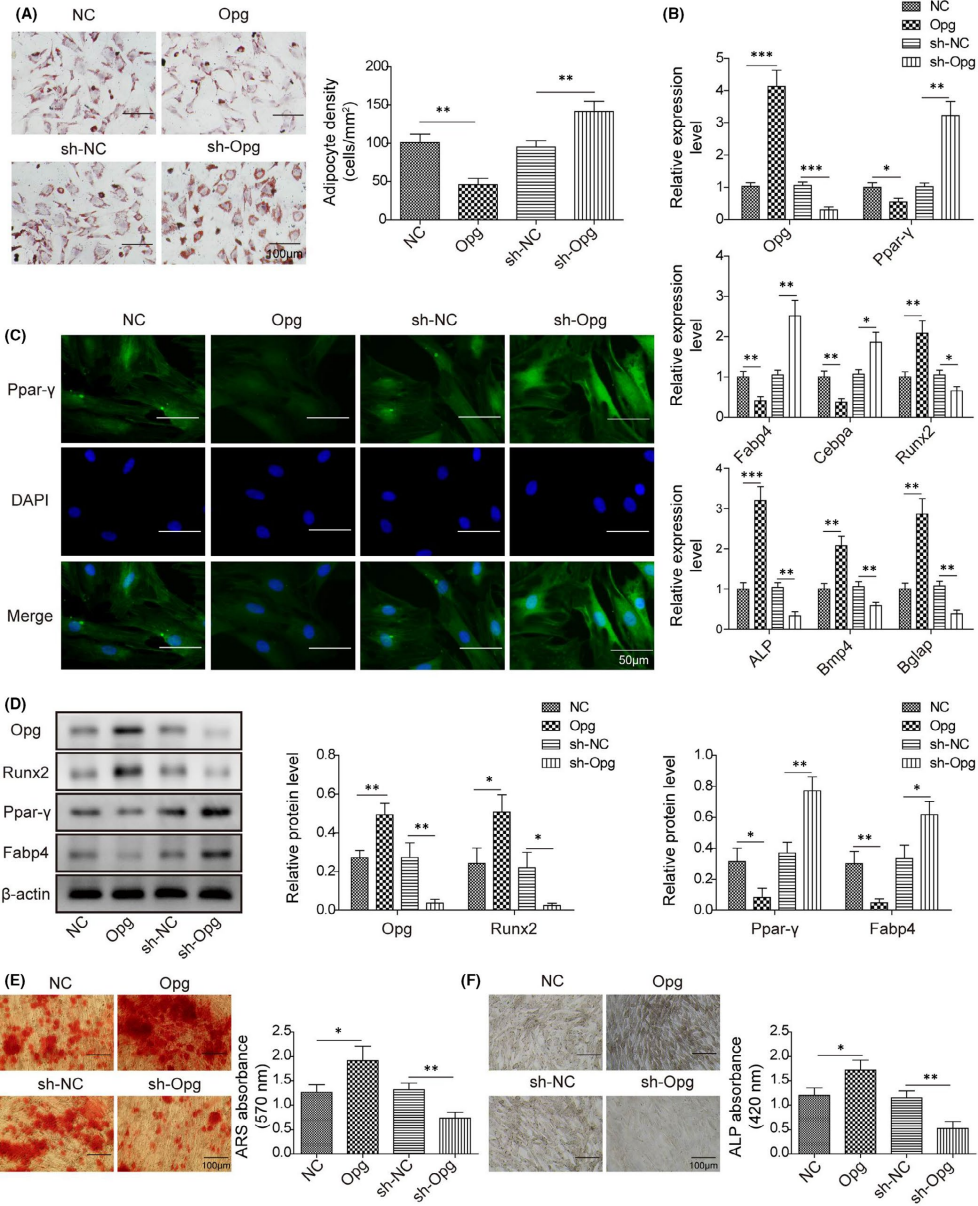
Opg regulates cell differentiation of BMSCs. BMSCs were transfected with pcDNA‐Opg/empty vector or sh‐Opg/sh‐NC. (A) Calculated adipocyte density after ORO staining with represented images; (B) The mRNA expressions of Opg, Runx2, Ppar‐γ, Fabp4, ALP, Bmp4, Bglap and Cebpa by qPCR; (C) Immunofluorescence staining for Ppar‐γ; (D) The protein levels of Opg, Runx2, Ppar‐γ and Fabp4 by western blot; (E) ARS absorbance after ARS staining with represented images; (F) ALP absorbance after ALP staining with represented images. Comparisons were conducted using one‐way ANOVA with post‐hoc analysis. *p < 0.05; **p < 0.01; ***p < 0.001

The role of Opg in BMSC differentiation was examined in BMSCs treated with osteogenic or adipogenic inducing media for 14 days. Consistently, Opg overexpression markedly decreased lipid droplet formation and the gene and protein expressions of adipogenesis‐related genes (Ppar‐γ, Fabp4 and Cebpa), but increased mineralized nodule formation, ALP activity, and the expressions of osteogenesis‐related genes (Opg, Runx2, ALP, Bmp4 and Bglap) (Figure 7A–F). All those effects were reversed when Opg was knocked down. Those results indicate that Opg regulates BMSC differentiation in a way that inhibits adipogenesis but promotes osteogenesis.

### 3.8 LncRNA SNHG1 silencing ameliorates osteoporosis in vivo

**FIGURE 8:**
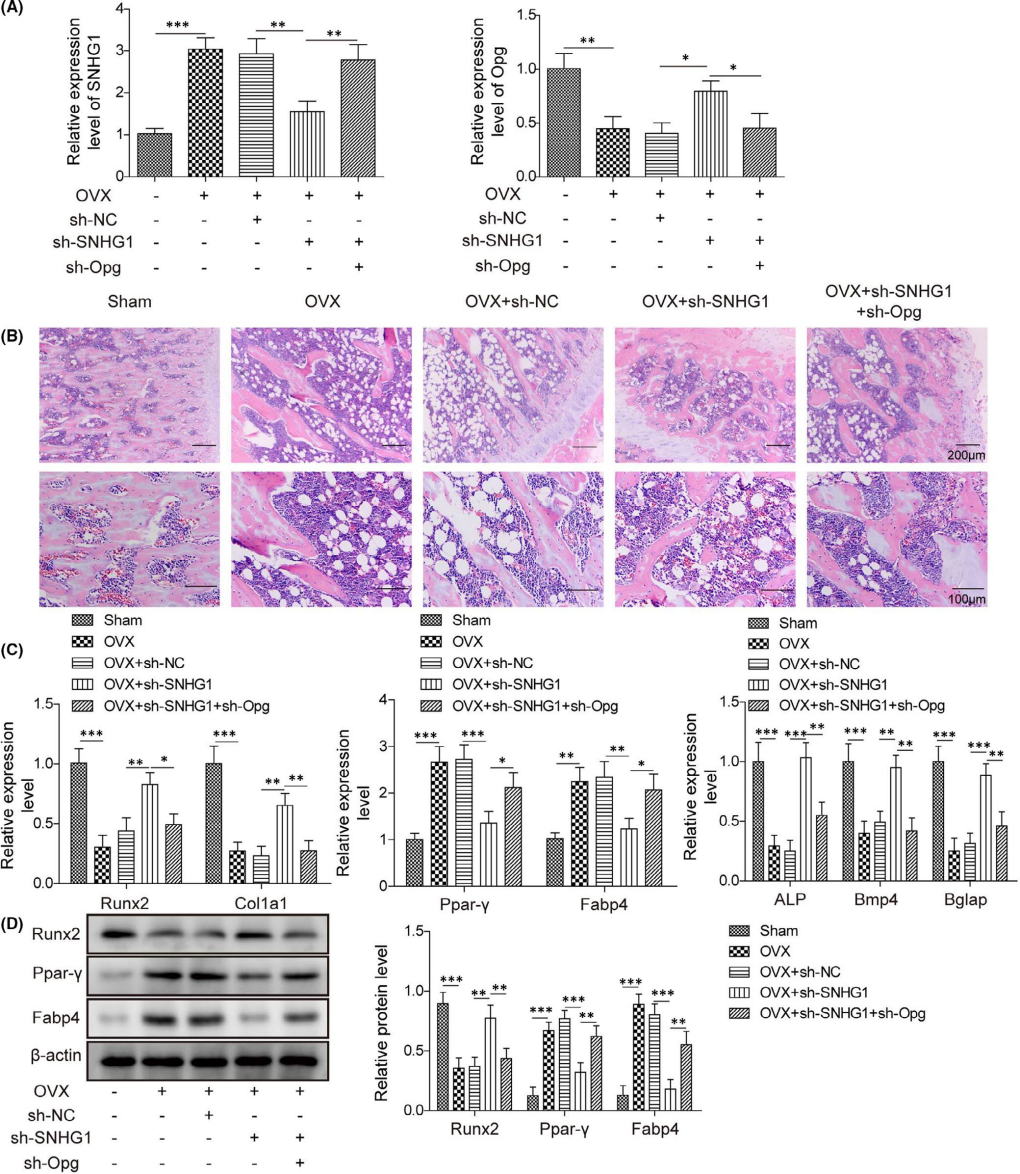
LncRNA SNHG1 silencing ameliorates osteoporosis in vivo. OVX mice were treated with sh‐SNHG1 and/or sh‐Opg via tail vein for 3 weeks. (A) The mRNA expressions of SNHG1 and Opg by qPCR; (B) Haematoxylin and eosin staining of mouse femurs; (C) The mRNA expressions of Col1a1, Runx2, Ppar‐γ, ALP, Bmp4, Bglap and Fabp4 by qPCR; (D) The protein levels of Runx2, Ppar‐γ and Fabp4 by western blot. Comparisons were conducted using one‐way ANOVA with post‐hoc analysis. *p < 0.05; **p < 0.01; ***p < 0.001

The in vitro studies we conducted suggest that lncRNA SNHG1 suppresses Opg expression via affecting the methylation status of Opg, resulting in enhanced BMSC adipogenic differentiation, which may contribute to osteoporosis. To confirm this, we further examined the effects of lncRNA SNHG1 in an in vivo mouse model of osteoporosis. Transfections with sh‐SNHG1 or sh‐Opg successfully repressed the expression of SNHG1 or Opg (Figure 8A). HE staining of mouse femur further confirmed osteoporosis in the OVX group, in which trabeculae was malalignment and the number and thickness were diminished, while trabecular separation was decreased as previously described^31^ (Figure 8B). Transfections with sh‐SNHG1 significantly improved osteoporotic changes in the OVX group, while co‐transfection with sh‐Opg partially reversed the effect of SNHG1 silencing (Figure 8B). In addition, qPCR revealed that SNHG1 silencing markedly upregulated the gene expressions of osteogenesis‐related genes (Runx2, ALP, Bmp4, Bglap and Col1a1) but downregulated the gene expressions of adipogenesis‐related genes (Ppar‐γ and Fabp4) (Figure 8C). Western bolt showed consistent results on the protein levels of those genes (Figure 8D). Thus, we conclude that SNHG1 plays a role in the osteoporotic changes in the in vivo model of osteoporosis, which may be mediated by Opg.

## 4 DISCUSSION

As one of the epigenetic regulators, lncRNAs play important roles in gene expression and multiple biological processes.^32^ Several studies suggest that lncRNAs are implicated in bone remolding by affecting the proliferation and function of osteoblasts and osteoclasts, as well as the differentiation of BMSCs.^33^ For example, lncRNA ANCR had been demonstrated to inhibit osteoblast differentiation and was essential to maintain osteoblasts in an undifferentiated state.^34^ On the contrary, lncRNA H19 promoted osteoblast differentiation of BMSCs, as evidenced by increased expressions of Runx2 and ALP.^9^ In the present study, lncRNA SNHG1 was found to enhance BMSC adipogenic differentiation but inhibit BMSC osteogenic differentiation, implicating its role in osteoporosis. Indeed, SNHG1 has been known to be a novel oncogenic lncRNA aberrantly expressed in a number of cancers and was linked to cell growth, migration and invasion.^35^, ^36^ Well agreed with our observations, lncRNA SNHG1 was previously reported to inhibit osteogenic differentiation of BMSCs through negatively modulating p38 MAPK signal pathway.^11^ Herein, lncRNA SNHG1 could be an interesting target for research on osteoporosis contributed by a shift of BMSC differentiation to adipocytes rather than osteoblasts.

Several lncRNAs have been reported to bind RBPs to regulate gene expression via cooperative or competitive interaction.^37^ PTBP1 is an RBP involved in all steps of RNA biogenesis and is implicated in metabolic and cancerous diseases via interacting with lncRNAs, such as H19, HCG22 and MACC1‐AS1.^38^, ^39^, ^40^ The interaction between lncRNA SNHG1 and PTBP1 had also been confirmed by RNA pull‐down and immunoprecipitation assays in our study. Moreover, by interacting with PTBP1, lncRNA SNHG1 upregulated the expression of DNMT1, which in turn promoted Opg hypermethylation. DNA methylation through adding a CH3 methyl group to cytosine by a DNMT is considered a long‐term, relatively stable epigenetic modification, resulting in inhibition of transcription and downregulation of target genes.^41^ In this regard, accumulating evidence suggested that the methylation status of Opg probably functioned as a “main switch” in the pathogenesis of osteoporosis.^19^, ^42^ The observations of Behera et al.^43^ that increased DNMT1 expression and hypermethylation of the Opg gene in a metabolite‐induced mouse model of osteoporosis further support the role of DNA methylation in the modulation of Opg expression in osteoporosis.

The regulatory function of Opg on BMSC differentiation and adipogenesis has also been suggested by a series of studies on transgenic animals.^44^, ^45^ In Opg knock‐out mice, mice developed early‐onset osteoporosis characterized by increased trabecular porosity and adipocyte accumulation in the bone marrow,^17^ while administration of Opg protein effectively reversed the osteoporotic bone phenotype presented in Opg‐deficient mice.^44^ Well agree with that, we found that Opg overexpression inhibited adipogenesis but promoted osteogenesis in BMSCs than cells transfect with control vector. Interestingly, we also found that lncRNA SNHG1 regulated the expression of Opg in osteoporotic mice; knockdown of SNHG1 improved osteoporotic changes, while simultaneous knockdown of Opg could partially reverse the beneficial effects of SNHG1 silencing on the loss of bone mass. However, in our study, there was lack of comparisons of gene and protein expressions during the duration of treatments. Additionally, MicroCT (or µCT) analysis was not performed to support the function of SNHG1 in osteoporosis. These were limitations of the present study.

In sum, our study demonstrated that lncRNA SNHG1 upregulated the expression of DNMT1 via interacting with PTBP1, resulting in Opg hypermethylation and decreased Opg expression, which in turn enhanced BMSC adipogenic differentiation and contributed to osteoporosis. This study provides additional insights into the molecular mechanisms of the lncRNA SNHG1/PTBPT1/DNMT1/Opg pathway in the pathogenesis of osteoporosis and presents novel ideas for developing new prevention and treatment measures for osteoporosis.

## CONFLICT OF INTEREST

The authors confirm that there are no conflicts of interest.

## AUTHOR CONTRIBUTION

**Xiao Yu:** Funding acquisition (equal); Supervision (equal); Writing‐original draft (equal). **Meng‐Sheng Song:** Data curation (equal). **Peng‐Ze Rong:** Investigation (equal); Validation (equal). **Xian‐Jun Chen:** Methodology (equal); Visualization (equal). **Lin Shi:** Formal analysis (equal). **Cheng‐Hao Wang:** Conceptualization (equal); Writing‐original draft (equal). **Qing‐Jiang Pang:** Methodology (equal); Writing‐review & editing (equal).

## ETHICAL APPROVAL

All animal procedures were performed in accordance with the guidelines by the National Institutes of Health Guide for the care and use of laboratory animals. All protocols were approved by the Animal Ethics Committee of our hospital. The clinical study was approved by the Ethics Committee of our hospital, and written informed consent was obtained from all participants.

## Data Availability

All data generated or analysed during this study are included in this published article.
